# Hyperoxia activates ATM independent from mitochondrial ROS and dysfunction

**DOI:** 10.1016/j.redox.2015.04.012

**Published:** 2015-05-02

**Authors:** Emily A. Resseguie, Rhonda J. Staversky, Paul S. Brookes, Michael A. O’Reilly

**Affiliations:** a Department of Environmental Medicine, University of Rochester, Rochester, NY 14642, USA; b Department of Pediatrics, University of Rochester, Rochester, NY 14642, USA; c Department of Anesthesiology, University of Rochester, Rochester, NY 14642, USA

**Keywords:** ATM, DNA damage, Hyperoxia, Mitochondrial dysfunction, OxPhos, Reactive oxygen species, ATM, ataxia telangiectasia mutated, DCF, 2′,7′-dichlorodihydrofluorescein diacetate, DHE, dihydroethidium, ETC, electron transport chain, mtDNA, mitochondrial DNA, PIKK, phosphatidylinositol 3-kinase-like kinase, ROS, reactive oxygen species, SMG-1, suppressor of Morphogenesis of Genitalia-1

## Abstract

High levels of oxygen (hyperoxia) are often used to treat individuals with respiratory distress, yet prolonged hyperoxia causes mitochondrial dysfunction and excessive reactive oxygen species (ROS) that can damage molecules such as DNA. Ataxia telangiectasia mutated (ATM) kinase is activated by nuclear DNA double strand breaks and delays hyperoxia-induced cell death through downstream targets p53 and p21. Evidence for its role in regulating mitochondrial function is emerging, yet it has not been determined if mitochondrial dysfunction or ROS activates ATM. Because ATM maintains mitochondrial homeostasis, we hypothesized that hyperoxia induces both mitochondrial dysfunction and ROS that activate ATM. In A549 lung epithelial cells, hyperoxia decreased mitochondrial respiratory reserve capacity at 12 h and basal respiration by 48 h. ROS were significantly increased at 24 h, yet mitochondrial DNA double strand breaks were not detected. ATM was not required for activating p53 when mitochondrial respiration was inhibited by chronic exposure to antimycin A. Also, ATM was not further activated by mitochondrial ROS, which were enhanced by depleting manganese superoxide dismutase (SOD2). In contrast, ATM dampened the accumulation of mitochondrial ROS during exposure to hyperoxia. Our findings suggest that hyperoxia-induced mitochondrial dysfunction and ROS do not activate ATM. ATM more likely carries out its canonical response to nuclear DNA damage and may function to attenuate mitochondrial ROS that contribute to oxygen toxicity.

## Highlights

• Hyperoxia induces mitochondrial ROS and respiratory dysfunction.
• Hyperoxia activates ATM-p53 signaling.
• Mitochondrial ROS and dysfunction do not activate ATM.
• ATM attenuates ROS accumulation during hyperoxia.

## Graphical abstract

**Figure:**
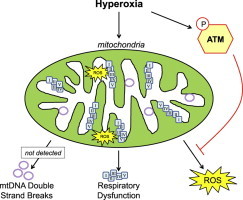

## Introduction

Oxygen is an essential element of cellular respiration, as it is the final electron acceptor in a process that generates ATP. During cellular respiration, mitochondria generate reactive oxygen species (ROS) when oxygen reacts with electrons that escape the electron transport chain (ETC) in the inner mitochondrial membrane. The free radical theory of aging proposes a vicious cycle in which mitochondrial ROS damage mitochondrial DNA (mtDNA), leading to improper coding of ETC proteins and dysfunction that further increases ROS [1]. While recent advances provide evidence against this theory [2], data still support an influential relationship between mitochondrial ROS, mtDNA damage, and respiratory dysfunction. MtDNA is thought to be quite susceptible to oxidative stress induced by mitochondrial dysfunction because it is in close proximity to the inner mitochondrial membrane [3]. To prevent oxidative stress and mtDNA damage under normal physiological conditions, mitochondrial ROS are quenched, in part, by the mitochondrial isoform of superoxide dismutase (SOD2), thioredoxin, peroxiredoxin and glutathione systems [4,5].

When antioxidant defenses become overwhelmed, oxidative damage can be detected in DNA, proteins and lipids throughout the cell. In response to nuclear DNA damage, ataxia telangiectasia mutated (ATM), a serine–threonine kinase, phosphorylates multiple targets to initiate DNA repair and cell cycle checkpoints [6,7]. One important ATM target is the tumor suppressor protein p53, whose phosphorylation on serine 15 induces its transcriptional activity towards genes such as *GADD45*, *BAX*, *P21*, and *PUMA* [8], thus controlling cell cycle progression, DNA repair and apoptosis. In addition to responding to nuclear DNA damage, ATM is involved in maintaining mtDNA copy number [9,10] and regulating respiration [11,12], although the mechanisms remain unclear. Likewise, p53 regulates mitochondrial respiration [13], and maintains mtDNA directly through interaction with polymerase gamma and indirectly as a nuclear transcription factor [14–16]. Even though both ATM and p53 are known to independently contribute to mitochondrial homeostasis, activation of the ATM–p53 pathway in response to mitochondrial damage has not been investigated.

Exposure to high oxygen (hyperoxia) provides a practical example of how ATM activation contributes to cell survival during oxidative stress. Supplemental oxygen is used to treat hypoxemia in premature infants, adults in acute respiratory distress and patients with Chronic Obstructive Pulmonary Disease (COPD). Although treatment with hyperoxia is often necessary to prevent mortality, persistent exposure to high oxygen can have long term effects on pulmonary function [17,18]. It is well established that hyperoxia induces oxidative damage, such as in lung epithelial [19,20] and endothelial [21,22] cells, that contributes to lung injury. Specifically, hyperoxia induces nuclear DNA damage [19,23,24] and has also been shown to induce 8-oxoguanine lesions localized to mitochondria in mouse and baboon lung epithelial cells [20,25]. Hyperoxia diminishes ETC function and alters mitochondrial morphology in animal and cell culture models [20,26,27]. ATM promotes cell survival late during hyperoxia by phosphorylating p53-Ser15, while early p53 phosphorylation is dependent on Suppressor of Morphogenesis of Genitalia-1 (SMG-1) [28,29]. p53 activation during hyperoxia is essential for stimulating expression of p21, a cyclin-dependent kinase inhibitor that mediates growth arrest and enhances cell survival [30].

Because mitochondrial function contributes to cell survival during hyperoxia exposure, it is important to understand the signaling pathways that respond to mitochondrial dysfunction. While ATM activation during hyperoxia is known to be protective, few studies have investigated whether it is activated specifically in response to mitochondrial dysfunction or ROS [31,32]. Therefore, we hypothesized that hyperoxia exposure activates ATM due to increased mitochondrial dysfunction and ROS. To address this hypothesis, we first assessed the timing of respiratory dysfunction, ROS, and mtDNA damage in human lung epithelial cells during hyperoxia. We then pharmacologically and genetically manipulated mitochondrial respiration and ROS to determine if mitochondrial dysfunction activates ATM.

## Materials and methods

### Cell culture

The human lung adenocarcinoma cell line A549 (American Type Culture Collection, Manassas, VA, USA) was cultured in Dulbecco’s modified Eagle medium (DMEM; high glucose) with 10% fetal bovine serum, 50 U/ml penicillin, and 50 µg/ml streptomycin (Gibco, Carlsbad, CA) in 5% CO_2_ at 37 °C. Cells were plated one day prior to exposure to room air or hyperoxia (95% O_2_, 5% CO_2_) in a Plexiglas chamber (Bellco Glass, Vineland, NJ). Cells were treated with KU55933 (Calbiochem/Millipore, Billerica, MA) or wortmannin (Sigma, Saint Louis, MO) for 1 h prior to hyperoxia exposure or treatment with Antimycin A (Sigma).

### Measurement of mitochondrial respiration

Both XF-24 and XF-96 Extracellular Flux Analyzers (Seahorse Biosciences, Billerica, MA) were used to measure the oxygen consumption rate (OCR) and the extracellular acidification rate (ECAR). When cells were plated on either polystyrene (PS) or polyethylene terephthalate (PET) XF plates prior to hyperoxia exposure, oxygen was absorbed by the plates and resulted in negative OCR measurements (unpublished observations). Therefore, A549 cells on normal tissue culture plates were exposed to hyperoxia, trypsinized at the end of the exposure, and all treatments were replated on a PS plate at 40,000 cells/well in 100 µl unbuffered DMEM pH 7.4 (1 mM sodium pyruvate, 4 mM l-glutamine, 25 mM dextrose) for the XF24, or 20,000 cells/well in 30 µl for the XF96, by centrifugation (40*g* for 1 s, reverse plate orientation, then 80*g* for 1 s, with slow acceleration and deceleration). The assay was initiated within 2 h of removal from hyperoxia. Basal respiration was measured before injection of 0.5 µM carbonyl cyanide-p-trifluoromethoxyphenyl-hydrazone (FCCP, Sigma) to stimulate maximal OCR. Non-mitochondrial OCR was measured after injection with 5 µM Antimycin A and subtracted from basal and maximal OCR measurements. Reserve capacity was calculated as the difference between maximal and basal OCR. Measurements were normalized to the number of cells per well for the XF24 assay, counted by hemacytometer after the experiment, or to protein determined by BCA assay (Thermo Scientific, Rockford, IL) for the XF96 assay.

### Measurement of mitochondrial mass

Cells were stained with 50 nM MitoTracker Green FM (Molecular Probes, Life Technologies, Eugene, OR) in 1× HBSS for 30 min at 37 °C. Using a BD LSRII 18-color flow cytometer (excitation 488 nm, emission 515 nm), 10,000 events gated as singlets (FSC-A/SSC-A then SSC-H/SSC-W) were collected for analysis. FlowJo software (FlowJo, Ashland, OR) was used to determine mean fluorescence intensity.

### Measurement of reactive oxygen species

Cells were stained with 10 µM carboxymethyl-H_2_-dichlorofluorescein diacetate (CM-H_2_DCFDA, Molecular Probes), 5 µM dihydroethidium (DHE, Molecular Probes), or 5 µM MitoSOX Red (Molecular Probes) in 1× HBSS for 15 min at 37 °C. Using a BD LSRII 18-color flow cytometer (DCF excitation 488 nm, emission 515 nm; DHE and MitoSOX excitation 532 nm, emission 575 nm), 10,000 events gated as singlets were collected for analysis. FlowJo software was used to determine mean fluorescence intensity.

### Southern blotting

After exposure to room air or hyperoxia, or treatment with bleomycin (Santa Cruz, Dallas, TX), cells were trypsinized, pelleted and stored at −20 °C until analysis. Total DNA was isolated using the DNeasy Blood & Tissue kit (Qiagen, Valencia, CA) according to the manufacturer’s instructions. Ten micrograms of DNA was digested with *BamHI* and subjected to gel electrophoresis on a 0.6% agarose gel without ethidium bromide. DNA was transferred to a nylon membrane (MSI MagnaGraph, 0.45 µM) using standard southern blotting technique [33]. A complimentary DNA fragment encoding the 12S and 16S rRNA region of mtDNA (5′-GGTCACACGATTAACCCAAG-3′ and 5′-GTTGGTTGATTGTAGATATTGG-3′) was radiolabeled using the Prime-a-Gene kit (Promega, Madison, WI) with α^32^P-dCTP and hybridized to the DNA on the blot overnight. Hybridization was visualized on blue x-ray film (Laboratory Products Sales, Rochester, NY).

### Immunoblotting

Cells were collected by scraping with a rubber policeman in lysis buffer as previously described [34]. Lysates were sonicated twice for 15 s at 20% with a stepped microtip (Sonics Vibra Cell, Newtown, CT). In separate experiments, cellular fractions were obtained using the Cell Fractionation Kit-Standard (Abcam, Cambridge, MA) according to the manufacturer’s instructions. Protein concentrations were determined using the BCA assay (Thermo Scientific) and extracts were diluted with 3× Laemmli Buffer (Laemmli at 1× contains 50 mM Tris (pH 6.8), 10% glycerol, 2% SDS, 1% β-mercaptoethanol, and 0.1% bromophenol blue). Samples were separated by SDS-PAGE, or on 4–15% Mini-PROTEAN TGX Stain-Free gels (BioRad, Hercules, CA) for p-ATM (Ser1981), and transferred to polyvinylidene difluoride (PVDF) membrane (Pall Life Sciences, Pensacola, FL). After blocking with 5% non-fat milk in TBS-T (0.1% Tween-20), blots were incubated overnight at 4 °C with primary antibody: ATM, P-p53 (Ser15), Histone H3 (Cell Signaling, Beverly, MA); p-ATM (Ser1981), SOD2 (Millipore); SOD1 (Santa Cruz); p53 (Novus, Littleton, CO); p21 (BD Pharmingen, San Jose, CA); Complex IV subunit 1 (Mitosciences, Eugene, OR); β-actin (Sigma). Secondary horseradish peroxidase (HRP) conjugated antibodies (Southern Biotech, Birmingham, AL) were incubated in 5% non-fat milk in TBS-T. Antibody complexes were detected using the ECL Plus Western Blotting Detection kit (GE Healthcare, UK) and visualized using X-ray film (Laboratory Products Sales). Blots were analyzed using ImageJ software (National Institutes of Health, Bethesda, MD).

### siRNA transfection

A549 cells at ~75% confluency were transfected with 100 nM non-targeting siGENOME siRNA (Dharmacon, Lafayette, CO), 100 nM ATM siRNA ([28], Dharmacon) or 10 nM SOD2 siRNA (s13269, Life Technologies) using Lipofectamine 2000 (Life Technologies) for 24 h, according to the manufacturer’s instructions. Cells replated at subconfluency were exposed to hyperoxia the following day.

#### Statistical analysis

**Fig. 1:**
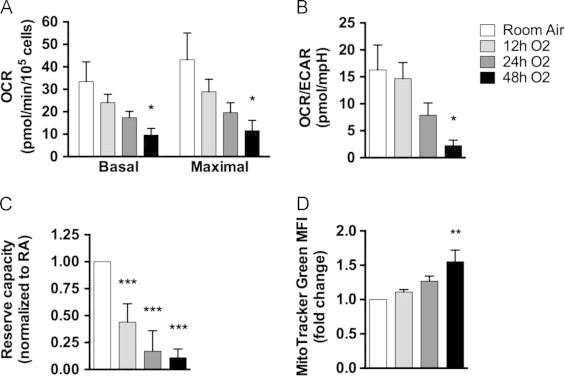
Hyperoxia induces mitochondrial dysfunction and increases mitochondrial mass. A549 cells were exposed to room air (RA) or hyperoxia (O_2_) for 12, 24 and 48 h. (A–C) After measuring the basal oxygen consumption rate (OCR) and extracellular acidification rate (ECAR), maximal OCR was stimulated with 0.5 µM FCCP. Respiration rates were normalized to the number of cells per well. (B) Bar chart depicts the ratio of basal OCR to ECAR. (C) Reserve capacity was calculated as the difference between basal and maximal OCR, normalized to RA within each experiment. (D) MitoTracker Green mean fluorescence intensity (MFI) was normalized to RA within each experiment. Data from 3 to 4 independent experiments are presented as mean±SEM (^⁎^*p*<0.05, ^⁎⁎^*p*<0.01, ^⁎⁎⁎^*p*<0.001 as compared to RA, ANOVA–Dunnett).

**Fig. 2:**
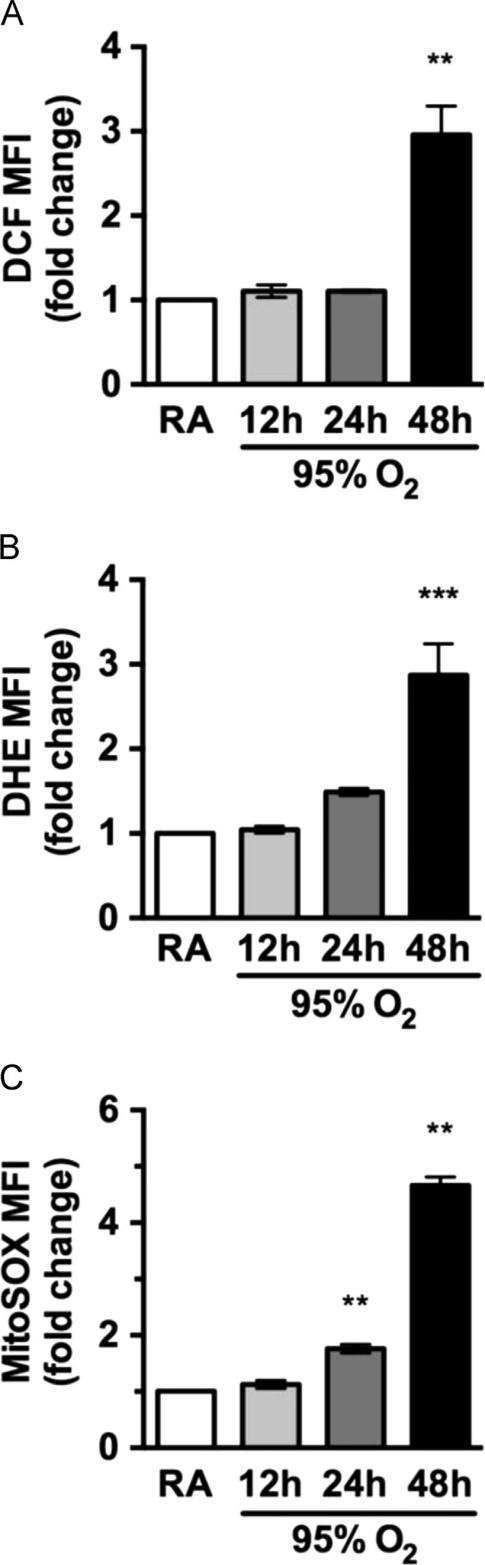
Hyperoxia increases reactive oxygen species late. Cells were exposed to room air (RA) or hyperoxia (95% O_2_) for 12, 24 and 48 h and then stained with CM-H_2_DCFDA (DCF, A) and DHE (B), or with MitoSOX-Red (C) prior to flow cytometry. Mean fluorescence intensity (MFI) was normalized to RA within each experiment. Data are presented as mean±SEM (^⁎⁎^*p*<0.01, ^⁎⁎⁎^*p*<0.001 as compared to RA, ANOVA–Dunnett, *n*=3).

Results represent at least three independent experiments and were graphed using Prism (GraphPad Software, La Jolla, CA). Group means were compared by ANOVA using JMP Software (SAS, Cary, NC). To control for multiple comparisons, Dunnett’s test was used to compare each hyperoxia time point to room air (Figs. 1 and 2), the Tukey–Kramer test was used when comparing different treatment groups (Fig. 5), and a Student’s *t*-test was used to compare groups within a given treatment (Fig. 6).

## Results

### Hyperoxia induces mitochondrial dysfunction and ROS

To determine when hyperoxia affects mitochondrial function in A549 human lung epithelial cells, the oxygen consumption rate (OCR) and extracellular acidification rate (ECAR) were simultaneously measured in cells exposed to room air or hyperoxia for 12, 24, and 48 h. A time dependent decrease in basal and maximal OCR occurred with hyperoxia exposure, which was significantly different compared to room air at 48 h (Fig. 1A). The concomitant increase in ECAR is reflected in the decreased OCR/ECAR ratio seen at 12–48 h of hyperoxia (Fig. 1B). Reserve capacity, or the ability of cells to up-regulate respiration in response to uncoupling stress, was not very large to begin with (compare basal and maximal OCR in Fig. 1A) and it was significantly diminished by 12 h and continued to decrease until 48 h in hyperoxia, when it reached 11% of the reserve capacity of room air cells (Fig. 1C). To assess changes in mitochondrial mass, cells were stained with MitoTracker Green. MitoTracker Green fluorescence increased over time in hyperoxia and was significantly elevated by 48 h (Fig. 1D), indicating that the loss of respiratory capacity was not due to a loss of mitochondrial mass.

Because mitochondrial respiratory dysfunction can augment production of superoxide, hyperoxia-induced changes in ROS were detected using several redox-sensitive fluorescent dyes. Carboxymethyl-H_2_-dichlorodihydrofluorescein diacetate (CM-H_2_DCF-DA, cleaved and oxidized to DCF) and dihydroethidium (DHE, oxidized mainly by superoxide to 2-hydroxyethidium) were used to detect general oxidative species and superoxide, respectively (see Kalyanaraman et al for review [35]). Significant increases in DCF and DHE fluorescence were found at 48 h of hyperoxia (Fig. 2A and B). Cells stained with MitoSOX, which detects mitochondrial superoxide, had increased fluorescence when exposed to 24 and 48 h of hyperoxia (Fig. 2C). Thus, the appearance of mitochondrial ROS lagged behind the decline in respiratory capacity.

**Fig. 3:**
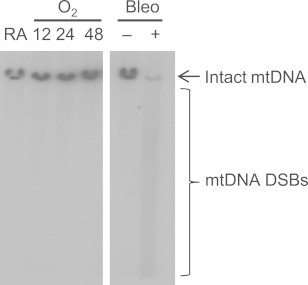
mtDNA double strand breaks are not detected in hyperoxia. Total DNA was collected from cells exposed to room air (RA) or hyperoxia (O_2_) for 12, 24 and 48 h, or treated with bleomycin (Bleo, 100 µM) for 24 h. Ten micrograms of DNA was digested with *BamHI* prior to neutral gel electrophoresis. Using Southern blot analysis, a 1.5 kb probe was used to detect mtDNA. mtDNA double strand breaks (DSBs) are visualized as smearing below the band of intact mtDNA. A representative blot is shown (*n*=3 for RA/O_2_, *n=*1 for Bleo).

Mitochondrial ROS are generated in close proximity to mtDNA and cause oxidative DNA damage, which can produce strand breaks [36]. Therefore, southern blotting was used to detect double strand breaks in mtDNA following hyperoxia exposure. Double strand breaks, visualized as a smear below the band of intact mtDNA, were not detected in cells exposed to 48 h hyperoxia (Fig. 3). Additionally, mtDNA double strand breaks were not detected in cells exposed for up to 96 h hyperoxia (data not shown). Bleomycin, an anti-cancer drug known to cause oxidative mtDNA damage [37,38], was used as a positive control. Mitochondrial DNA double strand breaks were, therefore, not detected at times when mitochondrial respiration declined and mitochondrial ROS increased.

### Hyperoxia activates ATM late during exposure

**Fig. 4:**
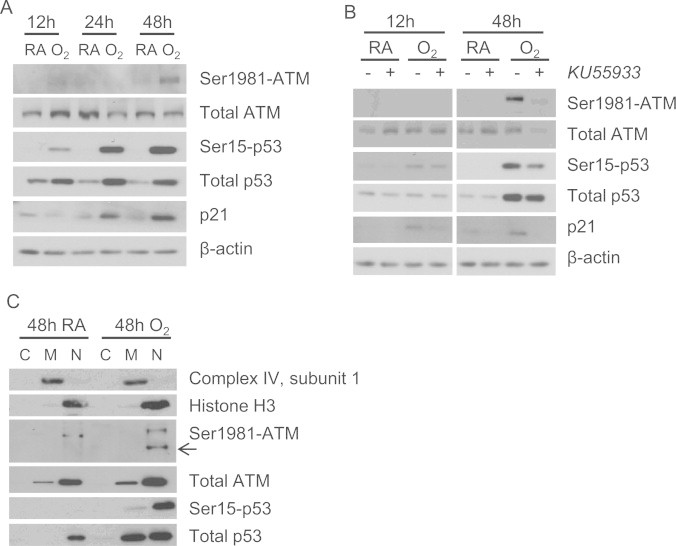
Hyperoxia induces nuclear ATM phosphorylation late. A549 cells were exposed to room air (RA) or hyperoxia (O_2_) and protein was collected 12, 24 and 48 h later. (A) Whole cell lysates were immunoblotted for phosphorylated and total ATM, phosphorylated and total p53, p21, and β-actin as a loading control. (B) Cells were pretreated with or without KU55933 (10 µM) 1 h prior to RA or O_2_ exposure, then whole cell lysate was collected after 12 and 48 h and immunoblotted as in (A). (C) Cellular fractions enriched for cytoplasmic (C), mitochondrial (M), and nuclear (N) proteins were immunoblotted for phosphorylated and total ATM, phosphorylated and total p53, with complex IV subunit I and histone H3 to assess purity of mitochondrial and nuclear fractions, respectively. Western blots are representative of at least 3 independent experiments.

Having established that hyperoxia induces a time-dependent decrease in mitochondrial respiration and increase in ROS, we then investigated ATM activation during these changes. Phosphorylated ATM-Ser1981 (p-ATM (Ser1981)), a marker of its activation, was detected at 48 h of hyperoxia (Fig. 4A). Hyperoxia did not affect ATM protein abundance, while phosphorylated p53-Ser15 (p-p53 (Ser15)) and its transcriptional target, p21, were increasingly detected over time (Fig. 4A). Consistent with ATM activation after prolonged hyperoxia exposure, pretreatment with KU55933, an ATM kinase-specific inhibitor [39], decreased p-ATM (Ser1981) at 48 h hyperoxia, which correlated with decreased total ATM, p-p53 (Ser15) and p21 (Fig. 4B). Cellular fractionation showed that p-ATM (Ser1981) localized in the nuclear fraction, while total ATM was found in both mitochondrial and nuclear fractions (Fig. 4C). Total p53 was detected in mitochondrial and nuclear fractions, while p-p53 (Ser15) was mainly nuclear with some detected in the mitochondrial fraction (Fig. 4C).

### Mitochondrial dysfunction and ROS production do not activate ATM

**Fig. 5:**
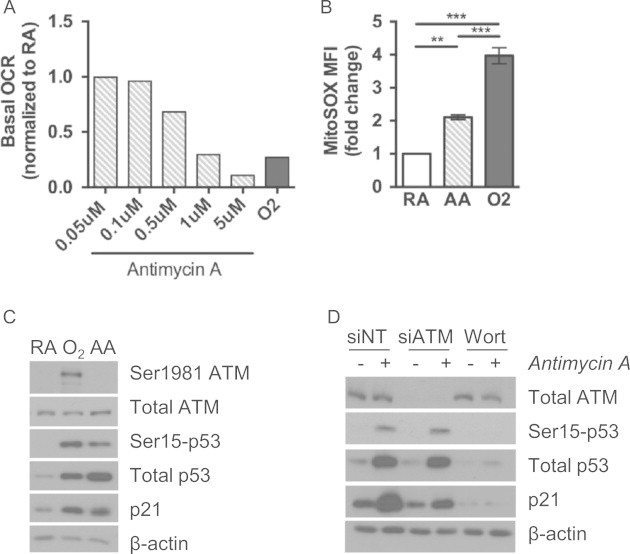
ATM phosphorylation is independent from mitochondrial dysfunction. Cells were exposed to room air (RA) or hyperoxia (O_2_), or treated with antimycin A (AA) for 48 h. (A) Basal oxygen consumption rates (OCR) were normalized to untreated RA cells (*n*=1). (B) After exposure to RA, O_2_, or 1 µM antimycin A, MitoSOX-Red mean fluorescence intensity (MFI) was normalized to RA within each experiment (*n*=3). (C) After exposure to RA, O_2_, or 1 µM antimycin A, whole cell lysates were immunoblotted for phosphorylated and total ATM, phosphorylated and total p53, p21, and β-actin as a loading control. (D) Cells were transfected with non-targeting (NT) or ATM siRNA, or pre-treated with wortmannin for 1 h, and exposed to 1 µM antimycin A. Whole cell lysates were immunoblotted as in (C). Bar chart and western blots are representative of 3 independent experiments (^⁎⁎^*p*<0.01, ^⁎⁎⁎^*p*<0.001 ANOVA–Tukey–Kramer).

Hyperoxia induced significant mitochondrial dysfunction and ROS at the time of ATM activation. To determine if ATM is activated by mitochondrial dysfunction during hyperoxia, a pharmacologic approach was taken. A chronic low dose of a Complex III inhibitor, antimycin A, was used to mimic the reduction in basal mitochondrial respiration seen with 48 h of hyperoxia. Based on a dose range of antimycin A, treatment with 1 µM was found to decrease basal respiration to the same extent as 48 h hyperoxia (Fig. 5A). This treatment also increased MitoSOX fluorescence compared to untreated cells, but to a lesser extent than 48 h of hyperoxia (Fig. 5B). Inhibiting mitochondrial respiration with antimycin A did not induce p-ATM (Ser1981), but did increase p-p53 (Ser15), total p53 and p21 at 48 h (Fig. 5C). Because multiple phosphatidylinositol 3-kinase-like kinases (PIKKs) can phosphorylate p53, cells were pretreated with 50 µM wortmannin, a nonspecific PIKK inhibitor. Wortmannin completely blocked the p-p53 (Ser15) and p21 induced by 48 h of antimycin A (Fig. 5D). However, transfection with ATM siRNA prior to treatment with antimycin A did not blunt p-p53 (Ser15) or p21 production after 48 h of antimycin A exposure (Fig. 5D).

**Fig. 6:**
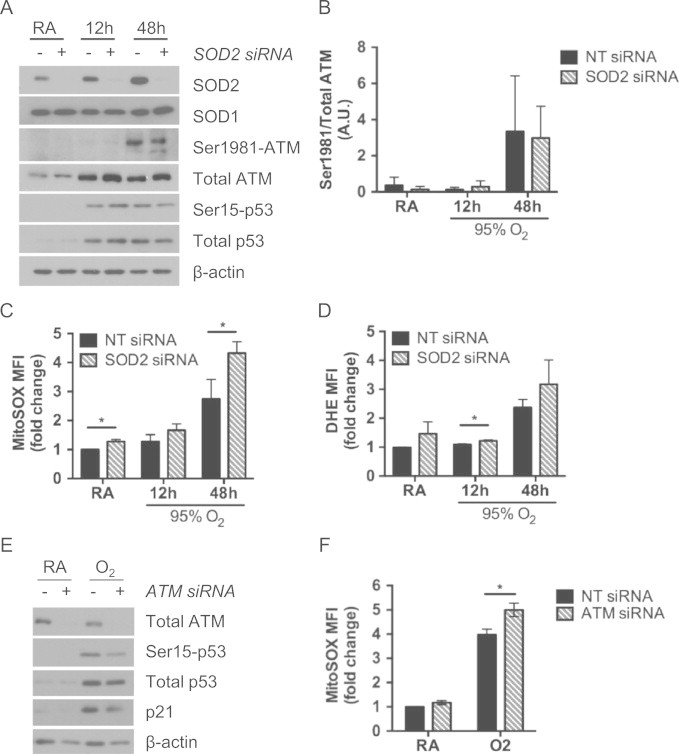
ATM deficiency enhances mitochondrial ROS but ATM phosphorylation is unaffected by mitochondrial ROS. Cells were transfected with non-targeting (NT), SOD2 or ATM siRNA prior to exposure to room air (RA) or hyperoxia (O_2_) for 12 and 48 h. (A) Whole cell lysates were immunoblotted for SOD2, SOD1, phosphorylated and total ATM, phosphorylated and total p53, and β-actin as a loading control. (B) Phosphorylated ATM was analyzed by densitometry and normalized to total ATM. Data are shown as mean±standard deviation (*n*=3). (C) MitoSOX-Red mean fluorescence intensity (MFI) and (D) DHE MFI of cells were normalized to room air NT siRNA within each experiment (^⁎^*p*<0.05 as compared to RA, ANOVA-*T*-test between NT and SOD2 siRNA). (E) Cells were transfected with NT or ATM siRNA and exposed to room air or hyperoxia for 48 h. Whole cell lysates were immunoblotted for total ATM, phosphorylated and total p53, p21 and β-actin as a loading control. (F) MitoSOX-Red MFI was normalized to room air NT siRNA within each experiment (^⁎^*p*<0.05 ANOVA–Tukey–Kramer). Western blots and bar charts are representative of at least independent 3 experiments.

To determine if ATM is activated by mitochondrial ROS during hyperoxia, cells were transfected with SOD2 siRNA to enhance oxidative stress during hyperoxia. Transfection with SOD2 siRNA diminished SOD2 protein abundance, without affecting SOD1 levels, and increased MitoSOX fluorescence in room air and hyperoxia, with minor increases in DHE fluorescence (Fig. 6A, C, and D). However, this did not affect hyperoxia-induced phosphorylation of p-ATM (Ser1981) or p-p53 (Ser15) at 12 or 48 h (Fig. 6A and B). Because ATM deficiency is associated with oxidative stress, we then determined if ATM influences mitochondrial ROS accumulation during hyperoxia. Transfection with ATM siRNA increased MitoSOX fluorescence in hyperoxia (Fig. 6F). As our laboratory previously published [28] and in agreement with the effects of KU55933 at 48 h (Fig. 4B), decreasing ATM expression resulted in less p-p53 (Ser15) during hyperoxia (Fig. 6E).

## Discussion

While functional mitochondria produce ROS during cellular respiration, exposure to hyperoxia causes mitochondrial dysfunction and increases ROS to cytotoxic levels. In response to hyperoxia, ATM-dependent p53 activation promotes cell survival. Because ATM and p53 contribute to maintaining mitochondrial homeostasis, this study explored whether hyperoxia activates ATM due to mitochondrial dysfunction and ROS. Using A549 lung epithelial cells, we show that hyperoxia decreases mitochondrial respiration and increases mitochondrial ROS at the time of ATM activation. Inhibiting mitochondrial respiration using antimycin A stimulated p53 phosphorylation that was independent of ATM but sensitive to wortmannin. Enhancing mitochondrial ROS by SOD2 suppression during hyperoxia did not affect ATM activation. Therefore, our data do not support the hypothesis that mitochondrial dysfunction and ROS activate ATM in hyperoxia. Instead, ATM attenuates the production of excessive ROS during hyperoxia in addition to responding to nuclear DNA damage.

Hyperoxia affects mitochondrial function in part by inhibiting aerobic respiration. Diminished basal OCR and reserve capacity have been noted in mouse lung mitochondria after 48–72 h hyperoxia [27,40], and in MLE12, A549, HeLa, and Chinese hamster ovary (CHO) cell lines after 24 h [26,27,41,42]. This has been attributed to decreased Complex I, II or IV enzyme activity, but not changes in protein abundance. Our results extend these findings by assessing cellular respiration after a shorter exposure to hyperoxia. Before a change in basal respiration is observed (Fig. 1A), A549 cells exhibit diminished reserve capacity after only 12 h of hyperoxia exposure (Fig. 1C), suggesting that hyperoxia may affect enzyme activity prior to reductions in basal aerobic respiration. In support of this idea, Gardner et al. [42] found that the activity of aconitase, a redox sensitive enzyme involved in the Krebs cycle, was decreased after 3 h of hyperoxia exposure in A549 cells. Less productivity by the Krebs cycle would limit the reducing equivalents available for oxidative phosphorylation. Our data also indicate that increased glycolysis compensates for decreased ATP production by oxidative phosphorylation (Fig. 1B). Cosentino et al. showed that ATM can promote glycolysis and production of NADPH by modulating glucose-6-phosphate dehydrogenase activity [43]. Decreased aerobic respiration is likely not due to a loss of mitochondria, as mitochondrial mass actually slightly increased during hyperoxia exposure (Fig. 1D). Diminished oxidative phosphorylation may be a result of redox changes or an initial attempt to minimize superoxide production that occurs during mitochondrial dysfunction [44].

Despite abundant evidence in the literature showing that ATM maintains mitochondrial homeostasis under normoxic conditions [11,31,43,45], there is limited evidence that mitochondrial dysfunction activates ATM. An acute lethal dose of carbonyl cyanide-m-chlorophenylhydrazone (CCCP) was shown to induce p-ATM (Ser1981) in mitochondria [10], yet this is not reflective of the gradual decline in mitochondrial function seen in hyperoxia. Antimycin A inhibits respiration and increases mitochondrial superoxide production by inhibiting electron transfer from ubiquinol to cytochrome *c* [46]. Treatment with low doses of antimycin A for 48 h induced a comparable reduction in basal respiration and induction of p-p53 (Ser15) as 48 h of hyperoxia, yet p-ATM (Ser1981) was not detected (Fig. 5). In contrast, p-p53 (Ser15) was not detected in cells treated with antimycin A for an acute period (data not shown), suggesting that the chronic reduction in basal respiration contributes to p53 activation. Cells with decreased ATM expression still expressed p-p53 (Ser15) following antimycin A (Fig. 5), indicating that ATM may be activated independent of mitochondrial dysfunction during hyperoxia. While this study focused on ATM-dependent p53 activation, pre-treatment with wortmannin, a non-specific PIKK inhibitor, implicates other kinases in mediating the response to mitochondrial dysfunction through p53 (Fig. 5). During hyperoxia, SMG-1 is responsible for p-p53 (Ser15) prior to ATM activation [28], yet SMG-1 does not mediate p53 phosphorylation during antimycin A treatment either (data not shown). Although antimycin A may not fully represent how hyperoxia suppresses mitochondrial respiration, it does show that ATM is not required for p53 activation during chronic inhibition of mitochondrial respiration.

Mitochondrial dysfunction may result in significantly increased ROS that cause damage throughout the cell. Several studies indicate that hyperoxia induces an initial burst of ROS that are presumably quenched until antioxidant defenses become overwhelmed after prolonged exposure [47,48]. Our results show that hyperoxia elevates mitochondrial ROS at 24 h, prior to increased whole cell ROS detected by DCF and DHE at 48 h (Fig. 2). The difference in timing could be explained either by actual differences in mitochondrial ROS levels compared to non-mitochondrial ROS [48], or by a difference in probe sensitivity, as MitoSOX is more positively charged than DHE due to the tetraphenylphosphonium targeting moiety [35]. It should be noted that the level of ROS that can cause oxidative damage may be below the limit of detection by MitoSOX. While hyperoxia increases mitochondrial superoxide, the relative importance of mitochondrial ROS in leading to cell death during hyperoxia is debated and may depend on the model system [44,49].

ATM deficiency is associated with increased oxidative stress, although the mechanism has not been elucidated. In support of ATM regulating oxidative stress, A549 cells transfected with ATM siRNA exhibited increased mitochondrial ROS during hyperoxia (Fig. 6). Because ATM siRNA efficiently knocked down protein abundance, the modest changes in MitoSOX fluorescence could be due to probe sensitivity or to the minor role of ATM in modulating ROS during hyperoxia. A recent study found NOX4, an NADPH oxidase that generates intracellular ROS, to be elevated in tissues affected by ATM deficiency [50]; however, NOX4 protein was not elevated in our studies with ATM siRNA (data not shown). ATM activation by oxidative stress can be observed independent from nuclear DNA double strand breaks [32,51]. A recent study showed that mitochondria were necessary to activate ATM in response to a dose of H_2_O_2_ that did not induce p-p53 (Ser15) or gamma-H2AX, a marker of nuclear DNA damage [32]. To investigate the role of ATM in responding to mitochondrial oxidative stress during hyperoxia, mitochondrial ROS levels were enhanced by depleting cells of SOD2. While SOD2 siRNA did not affect SOD1 protein abundance, other antioxidant systems may have compensated for limited superoxide dismutase activity and thus attenuated effects on MitoSOX and DHE fluorescence (Fig. 6). However, phosphorylated ATM and p53 were unaffected by the enhanced mitochondrial superoxide due to SOD2 depletion (Fig. 6). It is possible that ATM is phosphorylated in response to ROS besides superoxide, such as hydrogen peroxide and its metabolites [32], and unphosphorylated ATM may still respond to oxidative damage [51]. These data suggest that ATM may regulate ROS abundance, but that it is activated independent of increased mitochondrial ROS.

ATM deficiency is also associated with radiosensitivity and increased incidence of cancer, which are attributed to its role in responding to DNA damage [11,52]. ATM responds to nuclear DNA double strand breaks, which occur with 48 h of hyperoxia in A549 cells [23,53]. Here, we show for the first time that hyperoxia induces p-ATM (Ser1981) localizing in nuclear but not mitochondrial or cytoplasmic fractions at the time of ATM-dependent p53 phosphorylation (Fig. 4C). It is important to note that ATM auto-phosphorylation on Ser1981 is a marker of activation, although it is not necessary for kinase activity [51]. P-p53 (Ser15) was found in the mitochondrial fraction in addition to nuclear localization at 48 h (Fig. 4C), perhaps due to intracellular trafficking after nuclear activation, phosphorylation by un-phosphorylated ATM, or phosphorylation by another kinase such as SMG-1. As previously published [28,29], ATM knockdown lessened the induction of p-p53 (Ser15) at 48 h of hyperoxia (Fig. 6). Phosphorylation of p53 on serine 15 is known to be important for its nuclear transcriptional activity, yet its function in the mitochondria has not been established.

Nuclear DNA damage due to hyperoxia exposure has been characterized in more studies than mtDNA damage [23,24]; yet assessing mtDNA damage provides critical information for understanding how oxidative damage to this genome contributes to cell fate pathways. We recently attempted to elucidate ATM signaling in response to mtDNA damage by expressing a mitochondrial-targeted restriction endonuclease [54]. Although mtDNA double strand breaks were detected, the endonuclease also localized in the nucleus, precluding the definitive conclusion that ATM activation was strictly due to mitochondrial DNA damage. In a recent study that assessed DNA integrity in fetal rat lung explants, alkaline-sensitive mtDNA damage was detected after exposure to 21% oxygen (hyperoxia compared to in utero) for 24 h [55]. In contrast, replication-blocking lesions were not detected in pulmonary mtDNA of adult rats exposed to 48 h of hyperoxia [56]. Similarly, mtDNA double strand breaks during hyperoxia were not detected in A549 cells (Fig. 3). Hyperoxia-induced mtDNA double strand breaks may have been undetectable because of efficient repair, rapid degradation of damaged mitochondria, or because effects were diluted out by multiple mtDNA copies per cell. It is also possible that hyperoxia causes oxidative lesions that do not result in mtDNA double strand breaks [20,36]. However, this suggests that the decline in respiratory capacity and increase in ROS were not direct consequences of mtDNA damage due to high oxygen.

In conclusion, prolonged hyperoxia activates ATM independent of mitochondrial dysfunction and ROS accumulation. ATM may be involved in repressing ROS formation during hyperoxia, although further studies are needed to elucidate the mechanism. Moreover, our findings show that late p53 activation occurs apart from mitochondrial ROS induced by hyperoxia. Taken together, this study suggests that ATM promotes survival during hyperoxia in response to stimuli other than mitochondrial dysfunction and ROS, such as nuclear DNA double strand breaks that occur after prolonged exposure.
